# Characterizing the genomic variation and population dynamics of *Plasmodium falciparum* malaria parasites in and around Lake Victoria, Kenya

**DOI:** 10.1038/s41598-021-99192-1

**Published:** 2021-10-06

**Authors:** Ashley Osborne, Emilia Manko, Mika Takeda, Akira Kaneko, Wataru Kagaya, Chim Chan, Mtakai Ngara, James Kongere, Kiyoshi Kita, Susana Campino, Osamu Kaneko, Jesse Gitaka, Taane G. Clark

**Affiliations:** 1 Faculty of Infectious and Tropical Diseases, London School of Hygiene and Tropical Medicine, London, UK; 2 Department of Protozoology, Institute of Tropical Medicine, Nagasaki University, Nagasaki, Japan; 3 Department of Parasitology, Graduate School of Medicine, Osaka City University, Osaka, Japan; 4 Department of Microbiology, Tumor and Cell Biology, Karolinska Institutet, Stockholm, Sweden; 5 Centre for Research in Tropical Medicine and Community Development (CRTMCD), Hospital Road Next to Kenyatta National Hospital, Nairobi, Kenya; 6 School of Tropical Medicine and Global Health, Nagasaki University, Nagasaki, Japan; 7 Directorate of Research and Innovation, Mount Kenya University, Thika, Kenya; 8 Centre for Malaria Elimination, Mount Kenya University, Thika, Kenya; 9 Faculty of Epidemiology and Population Health, London School of Hygiene and Tropical Medicine, London, UK

**Keywords:** Genomics, Malaria

## Abstract

Characterising the genomic variation and population dynamics of *Plasmodium falciparum* parasites in high transmission regions of Sub-Saharan Africa is crucial to the long-term efficacy of regional malaria elimination campaigns and eradication. Whole-genome sequencing (WGS) technologies can contribute towards understanding the epidemiology and structural variation landscape of *P. falciparum* populations, including those within the Lake Victoria basin, a region of intense transmission. Here we provide a baseline assessment of the genomic diversity of *P. falciparum* isolates in the Lake region of Kenya, which has sparse genetic data. Lake region isolates are placed within the context of African-wide populations using Illumina WGS data and population genomic analyses. Our analysis revealed that *P. falciparum* isolates from Lake Victoria form a cluster within the East African parasite population. These isolates also appear to have distinct ancestral origins, containing genome-wide signatures from both Central and East African lineages. Known drug resistance biomarkers were observed at similar frequencies to those of East African parasite populations, including the S160N/T mutation in the *pfap2mu* gene, which has been associated with delayed clearance by artemisinin-based combination therapy. Overall, our work provides a first assessment of *P. falciparum* genetic diversity within the Lake Victoria basin, a region targeting malaria elimination.

## Introduction

Despite decades of research and elimination campaigns, malaria remains a major global health threat and is the sixth leading cause of death in low-income countries, with an estimated 228 million cases and ~ 405,000 deaths worldwide in 2019 alone^1^. The Sub-Saharan African region accounts for more than 93% of all cases and deaths, with children under five years old disproportionately affected^1^. *Plasmodium falciparum* is the most prevalent and deadly malaria parasite in sub-Saharan Africa, as well as the cause of almost all severe disease, including severe malarial anaemia and cerebral malaria^2,3^. Even with progress over the past few decades, reduction in malaria-associated deaths has decelerated since 2016 and progress towards global malaria eradication overall has slowed, in part due to the emergence and spread of drug resistance in malaria parasites and insecticide resistance in their mosquito vectors^1^. Due to their isolated geography and distinctive population dynamics, inhabited islands, such as those within the Lake Victoria basin, offer unique environments to test malaria elimination strategies^4^.

Studies in low transmission settings have provided valuable insight into malaria elimination strategies that may be viable for islands, as well as an increased understanding of the role human movement plays in malaria transmission in these environments^5^. The implementation of intensive and targeted measures on islands in low-transmission settings, including vector control based strategies (e.g. insecticide-treated nets and indoor residual spraying) and mass drug administration, has led to malaria elimination in places such as the Maldives and Reunion archipelagos^6^. Recently, attention has moved to islands in high-transmission regions where there is an increased rate of gene flow within and between malaria parasite populations, which may aid in propagating novel traits detrimental to malaria treatment outcomes and elimination campaigns^4^. Past failures of malaria elimination campaigns on high-transmission islands, such as Zanzibar and the Comoros archipelago off the coast of East Africa, have highlighted the need for a better understanding of the roles human and vector migration play in settings with intense transmission patterns, and their impact on parasite population genetics and structure^7^. The development of methodologies that allow for the amplification of parasite DNA from infections with low parasite density, alongside the reduction in costs associated with next-generation whole genome sequencing (WGS), has made investigating parasite genomic diversity in malaria endemic settings attainable^8,9^.

Malaria transmission and infection risk has decreased in Kenya and cases of the disease are generally not seen in high-elevation regions, such as Nairobi, due to the inability of mosquito vectors to survive at such altitudes. Low-elevation regions, however, such as the Lake Victoria basin and coastal regions along the Indian Ocean, still struggle with high rates of malaria transmission as they provide ideal breeding habitats for *Anopheles* mosquitoes^10,11^. Depending on the season, *P. falciparum* infection rates in the Lake Victoria basin can exceed 40% for those aged 2 to 10 years old^12^. Due to the size of Lake Victoria, and its location along three country borders with differing government policies concerning the implementation and funding of malaria control programmes, applying any targeted vector control in and around Lake Victoria has proven incredibly difficult^4,11^. There are multiple inhabited islands within the Kenyan territory of Lake Victoria, such as Mfangano island (population size 26,000) and Ngodhe island (population size 600–1000), which offer a unique opportunity to study malaria elimination strategies and changes in parasite genetic diversity.

To date, there has been limited research in the Lake Victoria region aimed at characterising the genomic diversity of the malaria parasites present. Previous research has assessed the genetic diversity through targeted genotyping of drug resistance genes or using microsatellite markers to gain insight into population dynamics^4^. However, such approaches underestimate the overall genetic variation of a population as only a small proportion of the genome is represented^13^. Consequently, the genomic diversity and population dynamics of malaria parasites from islands within the region remain largely unexplored. WGS technologies provide a means for generating a comprehensive picture of the epidemiology and structural variation of the *P. falciparum* populations within the Lake Victoria basin^8^. Here, to provide a baseline level of genomic diversity within the Lake region, we generated WGS data for two islands, as well as the mainland sub-county Suba District (population size: North 124,938; South 122,383) and compared the resulting variation to publicly available whole genome sequences, from Kenya and the wider African continent through the Pf3K project (https://www.malariagen.net/parasite/pf3k). Our analysis revealed that *P. falciparum* isolates from the Lake Victoria region of Kenya have distinct ancestral origins, with a high proportion tied to Central and East African lineages, and form a distinct sub-group within East Africa in population structure analyses. Known drug resistance biomarkers were observed in the Lake Victoria isolates at similar frequencies to those of East African parasite populations.

## Results

### Genome data and multi-clonality

A total of 940,191 high-quality SNPs were identified in the non-hypervariable regions of the *P. falciparum* genome*.* The final dataset comprised 784 isolates from 9 different countries in Africa (Supplementary Table S1), which included those collected in and around Lake Victoria (Mfangano island, Ngodhe island, and Suba District; N = 48); Kenya (Kilifi, Kisumu, and Kombewa, N = 134); East Africa (Tanzania and Uganda, N = 139); West Africa (The Gambia and Mauritania, N = 159); Central Africa (Cameroon, N = 98); South Central Africa (Democratic Republic of the Congo, N = 97); Southeast Africa (Madagascar and Malawi, N = 119). The samples from Lake Victoria were collected across two inhabited islands and one mainland site (Supplementary Table S1; Supplementary Fig. S1). The average number of pairwise SNP differences, a measure of genetic diversity, were broadly similar within locations around the Lake basin (Mfangano island 7900.8; Suba District 8708.1; Kisumu and Kombewa 9224.7).

Multi-clonality was measured using the *F*_*WS*_ metric, which assesses within-host diversity in relation to the local population diversity to characterise the risk of out-crossing/inbreeding, with monoclonal samples exhibiting “high” *F*_*WS*_ estimates (i.e. ≥ 0.95)^14,15^. In the samples collected from Mfangano and Ngodhe islands, a mean *F*_*WS*_ of 0.944 was observed, with 70.2% of samples exhibiting “high” *F*_*WS*_ estimates. When samples from Mfangano island and Ngodhe island were combined with samples from Suba District, a mean *F*_*WS*_ of 0.940 was observed (72.9% ≥ 0.95). Most samples from Mfangano (N = 19) and a handful of samples from Suba district (N = 9) were cultured in vitro prior to sequencing, which is consistent with the “high” *F*_*WS*_ estimates observed.

Isolates from Kisumu and Kombewa, Kenya had a mean *F*_*WS*_ of 0.777 (31.1% ≥ 0.95). The low proportion of samples with high *F*_*WS*_ scores is generally associated with a high degree of panmixis between the parasites in the population and a low population sub-structure. Samples from the Lake Victoria Region (i.e., Mfangano island, Ngodhe island, Suba District, Kisumu, Kombewa, and Muleba, Tanzania) and samples from East Africa (i.e., Kenya and Tanzania) had mean *F*_*WS*_ scores of 0.840 (48.1% ≥ 0.95) and 0.856 (51.5% ≥ 0.95) respectively.

### P. falciparum isolates from Lake Victoria form a distinct sub-group within East Africa

**Figure 1:**
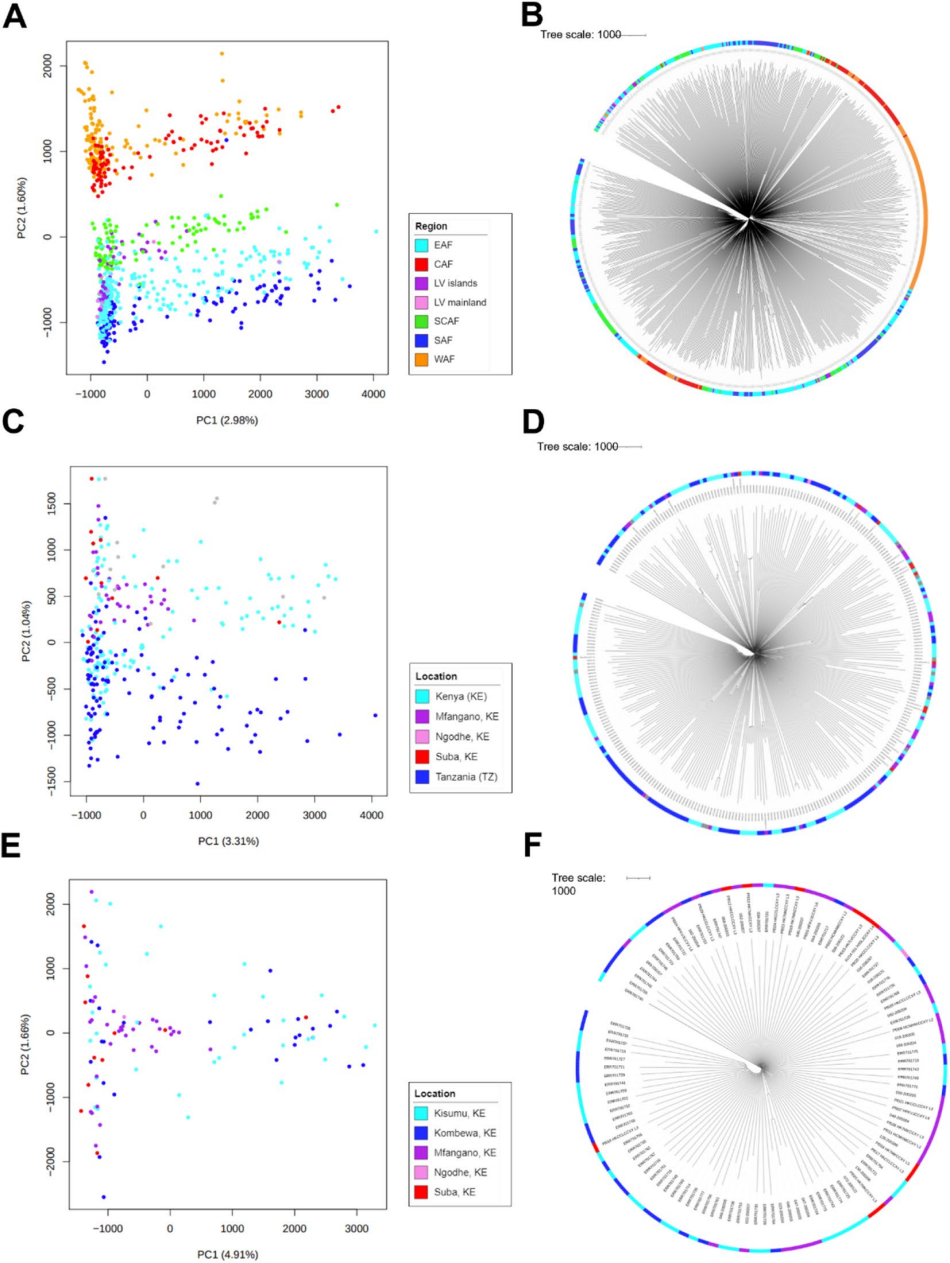
*Plasmodium falciparum* isolates from Lake Victoria (LV) form a distinct sub-group within East Africa. Principal component analysis (PCA) and neighbour-joining (NJ) trees generated from pairwise genetic distance matrices containing 940,191 quality filtered SNPs from 784 *P. falciparum* isolates. (**A**,**B**) A PCA plot and neighbour-joining tree for 784 isolates from East (EAF), Central (CAF), South Central (SCAF), Southeast (SAF), and West (WAF) Africa, LV mainland and islands. (**C**,**D**) A PCA plot and NJ tree for 321 isolates from EAF, LV mainland and islands. (**E**,**F**) A PCA plot and NJ tree for 109 isolates from Kenya, LV mainland and islands.

A SNP-based principal components analysis (PCA) and neighbour-joining tree revealed that Lake Victoria isolates (i.e., Mfangano island, Ngodhe island, Suba District, Kisumu, and Kombewa) cluster with the East African subpopulations when compared to larger regional African populations (Fig. 1A,B). To highlight the structure of the East African regional subpopulations, a SNP-based neighbour-joining tree and PCA were compiled using samples from Kenya, Tanzania, Uganda, and the Lake Victoria isolates. As anticipated, the Lake Victoria samples formed a sub-group within the larger East African dataset (Fig. 1C,D). A SNP-based neighbour-joining tree consisting of only samples from Lake Victoria provided a higher resolution of the population dynamics, and demonstrated that the isolates from the islands and Suba district appear to form a distinct genomic sub-group within the larger Lake Victoria population (Fig. 1E,F).

### Ancestral admixture analysis provides insight into the ancestral origins of Lake Victoria subpopulation

**Figure 2:**
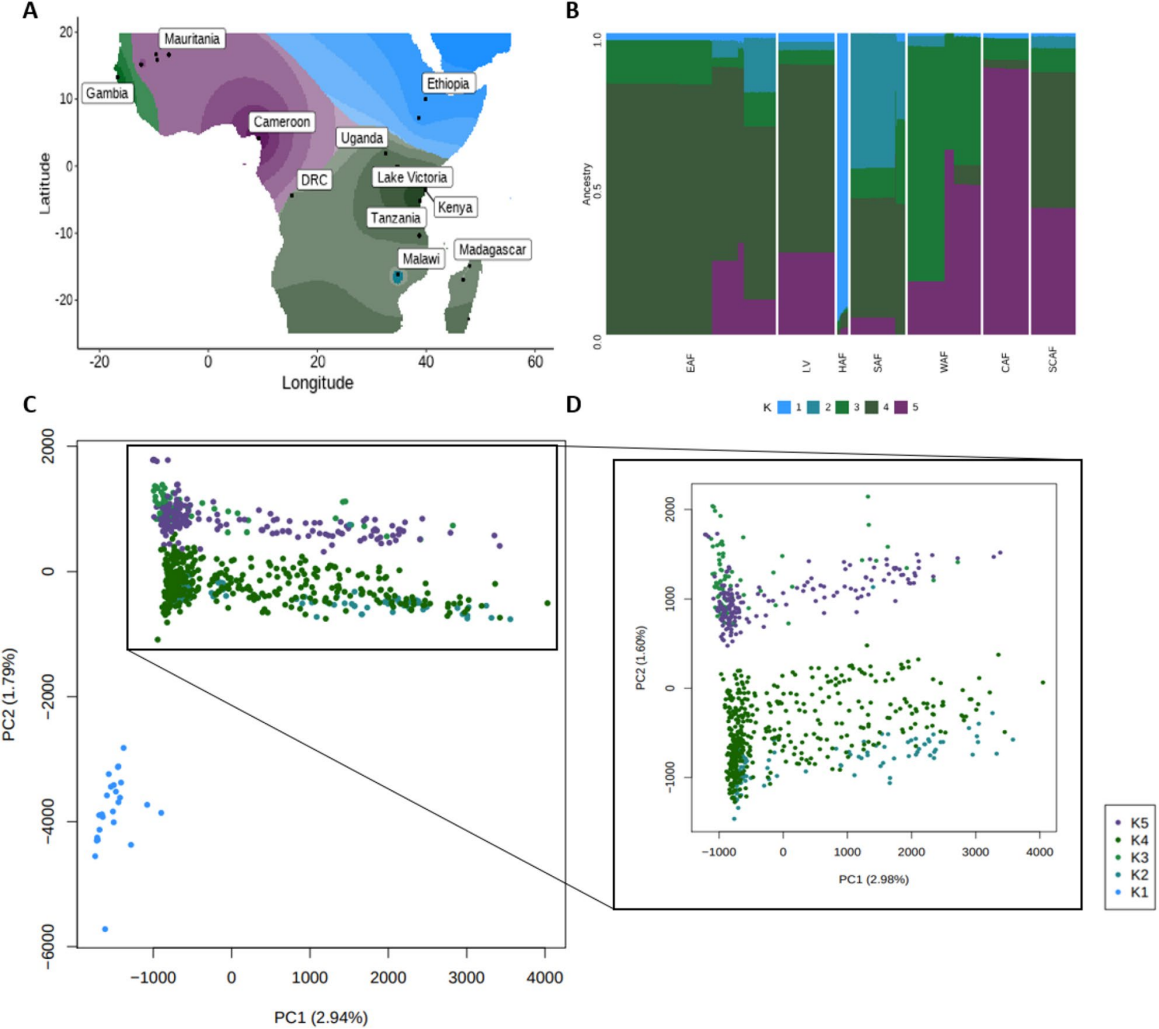
Genome-wide admixture ancestry proportions for regional *P. falciparum* populations across the African continent. (**A**) Geographic map of ancestry coefficients using *K* = 5 ancestral populations across Africa. (**B**) Ancestry per isolate (rows) for each regional population (columns). (**C**) Principal component analysis coloured using the Tess3r ancestry coefficients for the five predicted ancestral populations. (**D**) Cross-section of PCA excluding K1.

Spatial modelling of allele sharing using genome-wide SNPs and geographical coordinates was used to evaluate the ancestral origins of Lake Victoria isolates alongside the regional African populations. This ancestral admixture analysis revealed that Lake Victoria isolates contain differing proportions of ancestral genome pieces compared with East African isolates, where the optimum number of ancestral populations (K value) is estimated to be 5 (K1-K5) (Fig. 2). The K5 ancestral population appears to be linked to Central African isolates (Cameroon-like, 88.3%), K4 is linked to East Africa (Kenya-like, 83.0%; Tanzania-like 73.0%), and the K3 population appears to be linked to West Africa (Gambia-like, 78.0%). Lake Victoria isolates appeared to share a high proportion of their ancestral genome with East (K4, 62.3%) and Central (K5, 27.2%) African isolates. Further, a high proportion resemble Ugandan isolates, in addition to those from Kenya or Tanzania (Supplementary Fig. S1). As anticipated, the ancestry of Ethiopia, the Horn of Africa, was quite distinct compared to the other regional African populations, comprising mainly of the K1 ancestral population (92.7%), with limited ancestry in Lake Victoria isolates (2.9%).

### Analysis of IBD reveals differences within the Lake Victoria subpopulations

Analysis of identity-by-descent (IBD) was performed to understand the chromosome-level structure within subpopulations in and around Lake Victoria, Kenya. The proportion of pairs identical by descent at each SNP for all isolates was used to determine the extent of genomic relatedness. Isolates from Kisumu and Kombewa exhibited the highest fractions of pairwise IBD across the genome (Kisumu: median = 0.211, range = 0.182–0.238; Kombewa: median = 0.121, range = 0.069–0.200), reflecting high relatedness; while isolates from Mfangano and Suba exhibited lower fractions of IBD (Mfangano: median = 0.032, range = 0.018–0.142; Suba: median = 0.055, range = 0.055–0.132) (Supplementary Table S2). These lower fractions of IBD in the Mfangano and Suba isolates would suggest that relatedness is low, however their laboratory culture may be confounding these results as it is anticipated that samples in these populations would have high fractions of IBD across the genome. Examples of genome-wide IBD along each chromosome for individual sample sites and for regions across Africa are presented (Supplementary Fig. S2).

The top 5% of IBD positions in the island isolates from Lake Victoria were distributed across 31 regions on nine chromosomes ^1,3,5,6,7,8,11,12,and13^ (Supplementary Table S3). The region on chromosome 8 encompassed the *hydroxymethyldihydropterin pyrophosphokinase-dihydropteroate synthase* gene (*Pfdhps*, PF3D7_0810800), linked to Sulfadoxine–pyrimethamine (SP) antimalarial resistance. The top 5% of IBD positions in the mainland isolates from Lake Victoria were distributed across 13 regions on seven chromosomes ^3,6,7,8,12,13,and14^. While the top 5% of IBD positions in East African isolates included 35 regions across eight chromosomes ^3,4,5,6,7,8,12,and13^. The IBD region on chromosome 7 encompassed the *chloroquine resistance transporter* gene (*Pfcrt*, PF3D7_0709000), while the region on chromosome 8 included *Pfdhps* linked to SP resistance.

### Population differentiation between Lake Victoria isolates and others

Fixation index (*F*_*ST*_) analyses were performed to identify SNPs associated with the distinct subpopulations within the Lake Victoria region, and between other regional populations of the African continent. A genome-wide comparison of the differences in allele frequencies between samples collected from Suba District to those from Kisumu and Kombewa, identified 90 SNPs with an *F*_*ST*_ value of 0.5 or higher, suggesting some degree of genetic differentiation between these two subpopulations. In comparison, an analysis comparing Mfangano island with those from Kisumu and Kombewa identified 40 SNPs with an *F*_*ST*_ value greater than 0.5. A final comparison between samples collected from Mfangano island with those collected from Suba District resulted in 80 SNPs with an *F*_*ST*_ value of 0.5 or higher being identified. Across all island comparisons, most SNPs had an *F*_*ST*_ value of 0.5 or lower which suggests there is still frequent and consistent interbreeding between these populations. The lower number of significant SNPs for Mfangano comparisons may be explained by lower sequencing coverage leading to missing SNP genotypes, likely due to the lower parasite DNA concentrations often seen in the asymptomatic infections from which these samples were acquired. To identify genetic markers with the potential to be used to differentiate between isolates within the Lake Victoria region, and with populations across Africa, SNPs with an *F*_*ST*_ value of 1 (implying complete population differentiation) were compiled (Table 1). These may be used to determine the origin of a sample and monitor circulating parasites.

**Table 1:** Genetic markers for population differentiation between Lake Victoria isolates and other sub-populations according to the fixation index statistics (*F*_*ST*_). SNPs with an *F*_*ST*_ value of 1.

Chromosome	Position	Ref	Population 1 (allele)	Population 2 (allele)
14	2,468,958	A	Mfangano, Ngodhe & Suba (A)	Kisumu & Kombewa (G/AG)
6	67,235	T	Mfangano (A)	Kisumu & Kombewa (T)
14	2,468,958	A	Mfangano (A)	Kisumu & Kombewa (G/AG)
4	1,081,227	T	Suba (T)	Kisumu & Kombewa (G)
9	1,126,737	A	Suba (A)	Kisumu & Kombewa (T)
10	838,875	A	Suba (G)	Kisumu & Kombewa (A)
11	250,576	A	Suba (A)	Kisumu & Kombewa (G)
3	1,004,970	C	Mfangano (C)	Suba (T)
3	1,004,977	T	Mfangano (T)	Suba (C)
5	184,986	G	Mfangano (G)	Suba (A)
7	701,509	G	Mfangano (G)	Suba (A)
11	966,450	C	Mfangano (C)	Suba (T)
13	422,582	T	Mfangano (T)	Suba (G)
14	1,242,566	T	Mfangano (T)	Suba (A)
5	1,116,502	T	Lake Victoria (T)	Kilifi, Kenya (A)
6	59,375	T	Lake Victoria (T)	Kenya, Tanzania and Uganda (G)
6	59,378	C	Lake Victoria (C)	Kenya, Tanzania and Uganda (A)
14	2,468,951	A	Lake Victoria (A)	Kenya, Tanzania and Uganda (T)
5	1,116,502	T	Lake Victoria (T)	Cameroon (A)
6	68,360	C	Lake Victoria (T)	Cameroon (C)
14	2,468,958	A	Lake Victoria (A)	Cameroon (G/AG)
5	1,116,502	T	Lake Victoria (T)	Democratic Republic of Congo (A)
6	68,360	C	Lake Victoria (T)	Democratic Republic of Congo (C)
14	2,468,958	A	Lake Victoria (A)	Democratic Republic of Congo (G)
14	2,468,958	A	Lake Victoria (A)	Malawi and Madagascar (G)
6	68,229	T	Lake Victoria (A)	The Gambia and Mauritania (T)
14	2,468,951	A	Lake Victoria (A)	The Gambia and Mauritania (T)

Ref = Pf3D7 reference allele; *Lake Victoria = Mfangano, Ngodhe, Suba, Kisumu, and Kombewa; East Africa = Kenya, Tanzania, and Uganda; Central Africa = Cameroon; South Central Africa = Democratic Republic of Congo; Southeast Africa = Malawi and Madagascar; West Africa = The Gambia and Mauritania.

### Identification of mutations in drug resistance candidate genes

Non-synonymous SNP mutations in resistance-associated genes were identified in historical isolates from coastal (e.g., Suba district) and island parasite populations (e.g., Mfangano island) within Lake Victoria. The frequencies of these mutations in the Lake Victoria isolates were compared to frequencies observed in East African parasite populations (e.g., Tanzania and Kenya) and West African populations (e.g., Mauritania and The Gambia).

Resistance markers for chloroquine and SP were observed in the Lake Victoria isolates at similar frequencies seen in the East African populations, and slightly higher frequencies than in West African populations (Supplementary Table S4). The *Pfcrt* K76T mutation, often used as the main marker of chloroquine resistance, was observed in 17.9% (N = 29) of the Lake Victoria isolates, 16.7% (N = 228) of the East African isolates, and 13.0% (N = 159) of West African isolates. Mutations F938Y and D1246Y in *Pfmdr1* have been associated with resistance to chloroquine, with D1246Y linked to lumefantrine. These mutations were observed in low frequencies in the Lake Victoria (F938Y 3.6%; D1246Y 7.1%) and East African isolates (7.6%; 11.3%), but at higher frequencies in the West African populations (22.2%; 31.2%).

*Pfdhfr* mutations associated with SP resistance were observed at very high frequencies in both the Lake Victoria and East African isolates, with 100% of the Lake Victoria isolates containing the N51I and S108N mutations, compared to 93.3% and 99.5%, respectively, in the East African isolates. The C59R (Lake Victoria 92.9%; East Africa 87%) and I164L (Lake Victoria 7.1%; East Africa 2.2%) mutations were also present. The *Pfdhfr* mutations were observed at similar frequencies in the West African parasite populations, however the I164L polymorphism was not observed. The K540E mutation on *Pfdhps* associated with SP resistance was observed in 100% of the Lake Victoria isolates, higher than East (85.8%) and West (30.0%) African isolates.

No known mutations on *Pfk13* associated with artemisinin resistance were observed in the Lake Victoria or East African isolates. The S160N/T polymorphism on *Pfap2mu*, believed to be associated with delayed clearance by artemisinin combination therapies (ACTs), was observed in 17.8% (S160N) of the Lake Victoria isolates, 17.4% (S160N/T) of the East African isolates, and 38.0% (S160N) in West African isolates^16,17^.

### Regions under selection in Lake Victoria and other subpopulations

Analysis of haplotype structure within the Lake Victoria isolates was performed to determine genomic regions responding to positive directional selection, identified as having high local homozygosity relative to the neutral expectation. SNPs on genes under selective pressure in a distinct subpopulation were identified using the integrated haplotype score (*iHS*) test statistic, whereas cross-population selective pressures were identified by the *XP-EHH* metric, which contrasts extended haplotype homozygosity (*EHH*) profiles between populations (Supplementary Fig. S3, Supplementary Table S5, Supplementary Table S6).

As anticipated, the most common SNPs under selective pressure were those on genes associated with the host immune response and immune evasion. Within the Mfangano isolates there were 4 genes of interest identified with SNPs that had significant *iHS* values ((− log_10_[1–2|Φ_*iHS*_–0.5|]) > 4.0), including: *P. falciparum* apical membrane protein 1 (*Pfama1*), one of the leading malaria vaccine candidates, merozoite surface protein 3 (*Pfmsp3*), associated with erythrocyte invasion and host immunity, and the Peptidase family C50 which are associated with the pathogenicity of *P. falciparum* through parasite immune evasion or invasion of host cells (Supplementary Fig. S3, Supplementary Table S5). In isolates from Suba district, only a Plasmodium RNA of unknown function (RUF6) was identified to have significant *iHS* values. Genes of interest within isolates collected from islands in Lake Victoria (e.g., Mfangano and Ngodhe island) included C50, Plasmepsin X (PMX), a mediator of parasite egress and invasion, *Pfmsp3*, and *Pfama1*. *Pfama1* and *Pfmsp3* are vaccine candidates. There was no strong evidence of positive selection (*iHS* values < 4.0) in two other key candidates (reticulocyte-binding protein homologue-5 (*PfRH5*), circumsporozoite protein (*PfCSP*)).

Cross-population analysis of isolates from the Lake Victoria islands with isolates from the Lake Victoria mainland (e.g., Suba, district and Kisumu, Kenya; Muleba, Tanzania) identified 2 genes with significant *XP-EHH* values ((-log10[p-value]) > 5.0), PMX and the Duffy binding-like merozoite surface protein 2 (*Pfdblmsp2*) (Supplementary Fig. S3, Supplementary Table S6). Isolates from the Lake Victoria islands were also compared to isolates collected from East Africa (e.g., Kenya, Tanzania, and Uganda) and 3 genes were identified, including *Pfdblmsp2*, PMX, and a cell traversal protein for ookinetes and sporozoites (CelTOS) which is a conserved antigen believed to have protective potential. Isolates from the Lake Victoria islands and mainland were compared to West and Central Africa to assess regional differences in selection pressures. When compared to West Africa, PMX was found to have significant *XP-EHH* values in the Lake Victoria isolates. When isolates from the Lake Victoria mainland were compared to West Africa, 4 genes were identified to have significant *XP-EHH* values, including several genes associated with drug resistance, including pro-drug activation and resistance esterase (PARE; pepstatin), *Pfcrt* (chloroquine), and *Pfdhps* (SP), as well as CCR4-associated factor 1 (CAF1), an egress and invasion protein.

## Discussion

Advances in next-generation sequencing technologies have provided an increasingly viable means for exploring the genomic variation and population dynamics of malaria parasites in high-transmission regions, where reduction in incidence rates have slowed in recent years. Such platforms can generate comprehensive snapshots of a region’s epidemiology and structural variation^1,14,18^. Due to their isolated geography and distinctive population dynamics, islands in high-transmission regions offer a unique environment to study parasite gene flow and test malaria control strategies^4,5^. Here, we have provided a baseline level of genomic diversity within the Kenyan Lake Victoria basin that has revealed the presence of known drug resistance biomarkers. *P. falciparum* isolates from Lake Victoria form a cluster within the East African subpopulation, and island and Suba District isolates appear to form their own sub-group within the Lake Victoria basin. Further, the Lake Victoria parasite populations have central and east African lineage ancestry.

Low levels of genetic differentiation between the island sample sites are typically assumed to be due to the high levels of human traffic between the sampling sites. However, comparisons of isolates obtained from the Lake Victoria basin have highlighted distinct sub-grouping dynamics^4^. Although the movement of people and vectors is common around the islands and adjacent mainland, movement to and from other sections of the lake is limited by transportation infrastructure and geography. A handful of communities on the large island of Mfangano are somewhat isolated from communities with frequent migration to and from the mainland due to their rough terrain and limited access routes, which may account for some degree of differentiation between Mfangano parasites and those from the wider Lake Victoria basin.

Admixture analysis revealed insights into the ancestral origins of the Lake Victoria isolates within the wider African continent context. Lake Victoria isolates contained high proportions of ancestral genome fragments associated with Central African and East African populations. This analysis aligns well with the current prevailing *P. falciparum* origin hypothesis, suggesting that malaria first emerged in Central Africa before spreading out through early human migration, with subsequent more recent migration events throughout the African continent^19^. A closer look at the admixture ancestry of specific country populations demonstrated that isolates collected within the Lake Victoria basin (e.g., Kisumu, Kombewa, Mfangano, Ngodhe, and Suba from Kenya) appeared to share similar cumulative proportions of ancestral genome fragments as isolates from Uganda, but differed from the proportions observed in Kenyan and Tanzanian isolates. Future investigations with a greater density of sampling, including the integration of parasite and human genomics, could provide insights into the co-evolution of host–pathogen and be linked to ethnohistory.

Population differentiation (*F*_*ST*_) analysis identified SNPs specific to the Lake Victoria subpopulations and African regional populations. These SNPs have the potential to be utilised in a molecular surveillance tool to determine the main routes of transmission and parasite migration. Population-specific SNPs were identified within the characterised nuclear genomes and offer a high degree of specificity on a smaller spatial scale, generally to the country of origin. These markers could be combined with population-specific organellar SNPs, which provide regional or continental level resolution, as well as SNPs on merozoite surface protein genes (e.g. *Pfmsp1* and *Pfmsp2*), which can be used to assess transmission intensity^20–22^. Further sample collection and characterization of *P. falciparum* genome data from the Lake Victoria region, alongside a robust African dataset, would be needed to generate a panel of SNPs with the degree of resolution needed to generate an effective molecular surveillance tool.

Analysis of the haplotype structure in the Lake Victoria isolates identified significant SNPs under selective pressure on genes associated with the host immune response and parasite immune evasion, such as *Pfama1*, *Pfmsp3*, and PMX. *Pfama1* is a leading malaria vaccine candidate due to the essential role it plays in parasite invasion of erythrocytes. *Pfama1* and other vaccine candidate genes have been found to be under balancing selection^23^*.* PMX is another important mediator of parasite invasion and egress^24,25^. *Pfmsp3* is an attractive vaccine candidate due to its role in mediating the host immune response to Plasmodium parasites^26^. Cross-population analysis comparing parasite populations from the Lake Victoria islands with the local mainland and East African populations identified SNPs on genes associated with immune evasion, host immune response, and sporozoite traversal (e.g., *Pfdblmsp2*, PMX, CelTOS). Duffy-binding-like domains are associated with a variety of pathogenic phenotypes in *P. falciparum* and are attractive malaria vaccine candidates given their crucial involvement in erythrocyte invasion. While CelTOS is believed to mediate transmission to mosquito and vertebrate hosts^27,28^.

Analysis of identity-by-descent (IBD) revealed that isolates from Kisumu and Kombewa exhibited the highest fractions of pairwise IBD across the genome, reflecting high relatedness. Isolates from Mfangano and Suba exhibited lower fractions of IBD, which would suggest low relatedness between these isolates. However, given their close geographical proximity and the continuous movement of people between these sites, the culturing of some isolates prior to sequencing may be confounding these results^29^. The *Pfdhps* gene, known to be associated with resistance to SP, was found to be in the top 5% of IBD positions in island isolates from Lake Victoria, suggesting this gene is being highly conserved in this population, likely due to the continued use of SP as a method of intermittent preventative treatment in pregnancy (IPTp)^30,31^.

Malaria treatment strategies within this region and across the African continent have been tailored to preserve the efficacy of existing antimalarial drugs, while further reducing parasite incidence rates^32^. ACTs are the current first-line treatment for uncomplicated malaria in Kenya, with SP only used as an IPTp, in accordance with WHO guidelines^1,33^. Monotherapy treatments have been largely phased out, with artemisinin only used in combination therapies and chloroquine no longer available over the counter in pharmacies or as a first-line treatment. Despite its discontinued use, resistance markers for chloroquine resistance still persist in these Lake Victoria isolates and much of East Africa, apart from Malawi which has since seen the return of chloroquine sensitivity in its parasite populations following earlier changes to treatment policies^34,35^. It is anticipated that chloroquine sensitivity will return to parasite populations across Africa when drug selection pressure is completely removed, but the slow reduction in resistance marker frequencies suggests there is a low or negligent fitness cost associated with maintaining these polymorphisms.

A handful of resistance markers for SP were identified in the Lake Victoria isolates, with three established biomarkers (N51I and S108N on *Pfdhfr*; K540E on *Pfdhps*) observed to be almost fixed. The continued use of SP as an IPTp is likely providing enough driving pressure to maintain these polymorphisms within Lake Victoria and in parasite populations across the continent. However, given the high frequencies of these biomarkers, it is also possible that their presence does not come at a large fitness cost to the parasite. The S160N/T mutation in *Pfap2mu*, first documented in Kenyan children following delayed parasite clearance by ACT, was observed to be present at similar frequencies to the rest of East Africa and at a lower frequency than in West Africa^16^. Although further in vivo and in vitro studies are required to confirm the role polymorphisms in *Pfap2mu* play in resistance to ACTs, the presence of this mutation highlights the importance of monitoring parasite populations in high-transmission regions for the emergence of existing and novel resistance methods that could compromise the efficacy of ongoing malaria control and elimination strategies in Africa.

Due to the nature of working with field isolates, there were some limitations with this study. Most samples collected under this survey campaign are from asymptomatic individuals who generally have low parasitaemia levels, which often results in poor quality parasite DNA following extraction from blood spots. This issue was especially apparent in samples collected from Ngodhe island, where many cases of malaria are sub-microscopic and only detectable using PCR^12^. Due to these limitations, asymptomatic infections, in general, tend to be underrepresented in studies aimed at characterizing the genetic and genomic variation of malaria parasites, despite the fact they make up a majority of infections worldwide^36^. To overcome this, we applied selective whole genome amplification methodologies to select and amplify the parasite DNA in these field isolates^8,37,38^. This approach enabled us to increase parasite DNA concentrations in many samples that would normally not meet the requirements for whole genome sequencing, allowing us to provide the first baseline of the genomic variation and population dynamics of the Lake Victoria basin, with the potential for roll-out in other regions in Africa or globally.

Despite the progress made over the last decade, the reduction in malaria incidence rates and deaths has decelerated since 2016 and progress towards global malaria eradication has slowed. These trends, combined with setbacks to malaria control programmes, for example, due to the COVID-19 pandemic, have highlighted the need to generate comprehensive pictures of the epidemiology and structural variation of parasite populations. In high transmission regions, it is important to monitor the efficacy of current malaria control programmes and highlight potential challenges that may undermine their success. Here, we provided a baseline level of the genomic diversity of *P. falciparum* in the Lake Victoria basin of Kenya, a region whose genomic diversity has been previously unexplored. The application of sequencing technologies in malaria endemic regions will assist clinical management and disease control through surveillance activities. Known and putative drug resistance markers in established loci and population-specific markers are detectable using low-cost sequencing-based approaches (e.g., amplicon-based), suitable for a low resource setting. Large-scale approaches using (portable) whole genome sequencing and analysis will assist with understanding the transmission of malaria and provide insights into circulating drug resistance. Such insights will inform the deployment of antimalarial drugs and disease control tools and strategies in a region with currently high malaria burden, but with the potential for disease elimination.

## Materials and methods

### Study site selection

Parasite DNA sample collection for this study began in 2014 from several inhabited islands in Lake Victoria (Mfangano, N = 36; Ngodhe, N = 1) and the mainland sub-county of Homa Bay County, Suba district (Ungoye, N = 11) (Supplementary Fig. S4). Sample collection in this region was performed within an ongoing bi-annual survey, in accordance with the seasonal malaria prevalence. This seasonal transmission is linked to long (March–May) and short (October–December) rainy seasons. A peak in malaria prevalence is generally observed in June following the long rainy season and remains steady between September and February.

Permission to conduct this study was obtained from the Mount Kenya University Independent Ethics and Research Committee (MKU-IERC) (Approval reference: P609/10/2014) and the Ethics Committee at Osaka City University (Approval number: 3206) and performed in accordance with relevant guidelines and regulations. Workshops and sensitisation meetings were carried out with communities in order to seek community consent to study participation. Written informed consent was obtained from all study participants whose parasite DNA was used in this study.

### Study population characteristics

This region of the Lake Victoria basin is occupied by both Luo and Suba ethnic groups, with Ngodhe occupied entirely by the former. The Luo ethnic group are generally migrant fishermen, while the Suba ethnic group rely more heavily on subsistence farming^4^. The main mode of transportation on the lake are dugout boats, but a ferry service is also in place, operating regularly to bring merchants and workers between the islands and the mainland commercial hub, Mbita.

### Malaria species identification

Malaria species identification was carried out by microscopy, following WHO guidelines, at the Nagasaki University research station in Kenya by trained microscopists and confirmed at Osaka City University and LSHTM Malaria Reference Laboratory using established nested PCR assays^39,40^.

### Whole genome sequencing and bioinformatics

Twenty-nine isolates (collected years 2014 to 2015) were sequenced from DNA extracted from short-term (2–3month) cultures using Illumina MiSeq technology with 300 bp paired end kits organised by Nagasaki University. The DNA for a further twenty-nine isolates (collected year 2020) was extracted from filter papers and amplified using an established selective whole genome amplification (SWGA) primer set and protocols^8,41^, before being sequenced on Illumina MiSeq platform with 300 bp paired end kits at the LSHTM. All raw sequence data, following whole genome sequencing (WGS), was mapped to the Pf3D7 (*P. falciparum*) reference (version 3) genome using *bwa-mem* software (default parameters). SNPs and short insertions and deletions (indels) were called using the *samtools* and GATK software suites^28,29^. SNPs occurring in non-unique, highly variable (e.g., *var* genes), low quality or low coverage regions were discarded. Mixed call SNPs were assigned genotypes determined by a ratio of coverage in which nucleotide calls were 80% or higher. Samples with a coverage across the genome averaging less than 5 were not included in any analysis. Of the 58 samples sequenced, 10 were removed, leaving a total of 784 isolates in the final analyses.

### Characterising genomic variants and population genetic analyses

Population structure was investigated using neighbour-joining tree and principal component analyses, both using pairwise genetic distance matrices based on SNPs. Nucleotide diversity and population differentiation (*F*_*ST*_) metrics were calculated using the *pegas* R library^42^. The visualization and annotation of neighbour-joining trees was performed using *iTOL*^43^. The variants in drug-resistance genes were extracted from the sequence alignments, and annotated using *snpEff* software, which determined the type of mutation (e.g. non-synonymous, synonymous, or intergenic), as well as the codon and protein shift caused by any non-synonymous mutations and the projected impact of the polymorphism^44^. Grouping of samples into regional populations (e.g., Lake Victoria islands, Lake Victoria mainland, and East Africa) was determined based on patterns or human and vector migration within the region, alongside input from collaborators. WGS data from isolates collected on Mfangano island matched closely with the single isolate obtained from Ngodhe island, and were therefore combined into one population for further analysis.

The R-based *Tess3r* package, which estimates ancestry proportions by modelling on continuous genetic variation across space, was used to calculate admixture based on the spatial modelling of allele sharing using genome-wide SNPs and geographical coordinates from sampling sites (specified in the Pf3k dataset or recorded during sample collection for Lake Victoria isolates)^45–47^. An optimum K value for ancestral admixture coefficients was estimated as 5 from a cross-validation of 1 to 10 dimensions of eigenvalue decay. Bi-allelic sites were included in this analysis, while mixed calls were imputed to alternative and missing calls were left unchanged. The default spatial regularisation parameter (σ = 1) was used, which attributes equal weight to the loss and penalty functions. *Tess3r* was run 50 times for K5 to K10 and the best Q matrix of ancestry coefficients for every isolate was retained. This analysis used the alternating projected least squares algorithm (APLS; method = “projected.ls”). Plots were visualised using the R-based package *ggplot2* and surfaces were interpolated using the R-based package *Krig*^48–50^.

Multi-clonality, or within-host infection complexity, was measured by calculating the *F*_*WS*_ metric, using an in-house script, which assesses within-host diversity in relation to the local population diversity in order to characterise the risk of out-crossing/inbreeding by estimating the fixation of alleles, on a scale of 0 to 1, within each infection^14^. Based on previous studies, an *F*_*WS*_ ≥ 0.95 is considered highly indicative of a clonal infection.

Identity-by-descent (IBD) was used to assess connectivity between parasites within the Lake Victoria region and between subpopulations within the region. This was achieved by estimating the pairwise fraction of shared ancestry between genomic segments, the “IBD fraction”, that were inferred to have descended from a recent common ancestor without undergoing any intervening recombination. These IBD fractions were calculated using the *hmmIBD* software which accounts for recombination using a hidden Markov model-based approach^51^. The rate of recombination for *P. falciparum* is estimated to be 13.5 Kb per centiMorgan (cM), which should result in chromosomal crossover events occurring at an average rate of approximately 1% per generation.

Regions of the genome under putative positive directional selection were scanned using population-based measures of haplotype diversity within (iHS) or between (XP-EHH) populations using the R-based *rehh* package^52,53^.

## Supplementary Information

Supplementary Information.

## Data Availability

Public accession numbers for raw sequence data analysed are contained in SRA studies ERP000190 and ERP000199, as well as being accessible from the Pf3k project website (https://www.malariagen.net/projects/pf3k). Lake Victoria raw sequences are available from the EBI SRA (Project accession PRJEB46180) and DDBJ (BioProject Accession Number PRJDB12148).
