# Supplementary material for: Characterizing the genomic variation and population dynamics of Plasmodium falciparum malaria parasites in and around Lake Victoria, Kenya
Source: Sci Rep. 2021 Oct 6;11:19809. doi: 10.1038/s41598-021-99192-1 (PMC8494747; doi:10.1038/s41598-021-99192-1)

Supplementary Table S1. Summary data for *P. falciparum* isolates included in the SNP-based pairwise genetic distance matrices used to determine trends in population dynamics and identify genetic markers in sub-populations.

Country	Region	Number of samples
Kenya (Lake Victoria)	East Africa	48*
Kenya (other)	East Africa	134
Tanzania	East Africa	125
Uganda	East Africa	14
Madagascar	Southeast Africa	22
Malawi	Southeast Africa	97
Mauritania	West Africa	79
The Gambia	West Africa	80
Cameroon	Central Africa	98
Democratic Rep. of Congo	South Central Africa	97

***** Mfangano Island (36), Ngodhe Island (1), Suba District (11)

Supplementary Table S2. Fractions of pairwise identity-by-descent (IBD) across the genome.

Population	Median IBD	Range
Mfangano	0.032	0.018 – 0.142
Suba	0.055	0.055 – 0.132
Kisumu	0.211	0.182 – 0.238
Kombewa	0.121	0.069 – 0.200
Lake Victoria islands*	0.027	0.014 – 0.116
Lake Victoria mainland**	0.046	0.033 – 0.172
Kilifi, Kenya	0.040	0.024 – 0.204
Central Africa	0.042	0.030 – 0.132
East Africa	0.032	0.019 – 0.150
West Africa	0.040	0.025 – 0.210

*Lake Victoria islands (e.g., Mfangano and Ngodhe), ** Lake Victoria mainland (e.g., Suba, Kisumu, and Kombewa, KE), East Africa (e.g., Kilifi, Kenya; Muleba, Tanzania; Nachingwea, Tanzania; Apac, Uganda), Central Africa (e.g., Cameroon), West Africa (e.g., The Gambia and Mauritania)

Supplementary Table S3. Top 5% of identity-by-descent (IBD) regions in Lake Victoria isolates.

Chr	Start	End	Fraction	LV Category*	Location	Gene ID (PF3D7_)	Gene product	Gene name
1	530001	540000	0.015	Islands	537109 – 538025	0113900	CX3CL1-binding protein 1	*CBP1*
3	120001	130000	0.041	Islands	119458 – 124735	0302200	cytoadherence linked asexual protein 3.2	*CLAG3.2*
3	130001	140000	0.033	Islands	135418 – 140660	0302500	cytoadherence linked asexual protein 3.1	*CLAG3.1*
5	880001	890000	0.017	Islands	882376 – 884901	0521700	ATP-dependent RNA helicase DDX1, putative	*DDX1*
5	900001	910000	0.025	Islands	901414 – 902173	0522200	transcription initiation factor TFIID subunit 10, putative	*TAF10*
5	1010001	1020000	0.015	Islands	1013453 – 1014550	0524400	ribosome-interacting GTPase 1, putative	*RBG1*
6	1060001	1070000	0.017	Islands	1061115 – 1062891	0626300	3-oxoacyl-acyl-carrier protein synthase I/II	*FabB/FabF*
6	1070001	1080000	0.015	Islands	1078995 – 1081320	0626800	pyruvate kinase	*PyrK*
6	1080001	1090000	0.015	Islands	1078995 – 1081320	0626800	pyruvate kinase	*PyrK*
6	1110001	1120000	0.056	Islands	1114544 – 1117537	0627800	acetyl-CoA synthetase, putative	*ACS*
6	1200001	1210000	0.025	Islands	1205190 – 1207781	0629300	phospholipase, putative	*PL*
6	1230001	1240000	0.019	Islands	1221941 – 1242922	0629700	SET domain protein	*SET1*
6	1240001	1250000	0.031	Islands	1221941 – 1242922	0629700	SET domain protein	*SET1*
6	1250001	1260000	0.034	Islands	1254907 – 1256940	0630100	alpha/beta hydrolase	*N/A*
7	220001	230000	0.030	Islands	216024 – 229072	0704600	HECT-type E3 ubiq. ligase	*UT*
7	430001	440000	0.049	Islands	435089 – 436195	0709700	Prodrug activation and resistance esterase	*PARE*
7	470001	480000	0.018	Islands	478468 – 479138	0710600	60S ribosomal protein L34	*RPL34*
8	500001	510000	0.114	Islands	508224 – 512428	0809900	JmjC domain-containing protein, putative	*JmjC1*
8	510001	520000	0.116	Islands	508224 – 512428	0809900	JmjC domain-containing protein, putative	*JmjC1*
8	540001	550000	0.018	Islands	548200 – 550616	0810800	hydroxymethyldihydropterin pyrophosphokinase-dihydropteroate synthase	*PPPK-DHPS*
8	540001	550000	0.018	Islands	541971 – 544796	0810600	ATP-dependent RNA helicase DBP1, putative	*DBP1*
8	550001	560000	0.015	Islands	548200 – 550616	0810800	hydroxymethyldihydropterin pyrophosphokinase-dihydropteroate synthase	*PPPK-DHPS*
11	530001	540000	0.017	Islands	534973 – 536499	1113900	mitogen-activated protein kinase 2	*MAPK2*
11	550001	560000	0.016	Islands	555514 – 558668	1114700	cyclin-dependent-like kinase CLK3	*CLK3*
12	900001	910000	0.057	Islands	907203 – 914501	1222600	AP2 domain transcription factor AP2-G	*AP2-G*

12	910001	920000	0.057	Islands	907203 – 914501	1222600	AP2 domain transcription factor AP2-G	*AP2-G*
12	920001	930000	0.054	Islands	927825 – 929991	1223100	cAMP-dependent protein kinase regulatory subunit	*PKAr*
12	980001	990000	0.019	Islands	988628 – 991255	1224300	polyadenylate-binding protein 1, putative	*PABP1*
12	990001	1000000	0.029	Islands	998353 – 999275	1224500	histone chaperone ASF1, putative	*ASF1*
12	990001	1000000	0.029	Islands	988628 – 991255	1224300	polyadenylate-binding protein 1, putative	*PABP1*
13	100001	110000	0.024	Islands	99548 – 100521	1301700	CX3CL1-binding protein 2	*CBP2*
3	120001	130000	0.036	Mainland	119458 – 124735	0302200	cytoadherence linked asexual protein 3.2	*CLAG3.2*
6	1110001	1120000	0.152	Mainland	1114544 – 1117537	0627800	acetyl-CoA synthetase	*ACS*
7	470001	480000	0.038	Mainland	478468 – 479138	0710600	60S ribosomal protein L34	*RPL34*
8	410001	420000	0.054	Mainland	416344 – 418065	0808200	plasmepsin X	*PMX*
8	500001	510000	0.167	Mainland	508224 – 512428	0809900	JmjC domain-containing protein, putative	*JmjC1*
8	510001	520000	0.143	Mainland	508224 – 512428	0809900	JmjC domain-containing protein, putative	*JmjC1*
12	910001	920000	0.038	Mainland	907203 – 914501	1222600	AP2 domain transcription factor AP2-G	*AP2-G*
12	920001	930000	0.045	Mainland	927825 – 929991	1223100	cAMP-dependent protein kinase regulatory subunit	*PKAr*
12	970001	980000	0.039	Mainland	974372 – 975541	1224000	GTP cyclohydrolase 1	*GCH1*
12	980001	990000	0.034	Mainland	988628 – 991255	1224300	polyadenylate-binding protein 1, putative	*PABP1*
12	990001	1000000	0.036	Mainland	998353 – 999275	1224500	histone chaperone ASF1	*ASF1*
12	990001	1000000	0.036	Mainland	988628 – 991255	1224300	polyadenylate-binding protein 1, putative	*PABP1*
13	100001	110000	0.040	Mainland	99548 – 100521	1301700	CX3CL1-binding protein 2	*CBP2*
14	2700001	2710000	0.033	Mainland	2709418 – 2713192	1466300	26S proteasome regulatory subunit RPN2, putative	*RPN2*

*Lake Victoria islands (e.g., Mfangano and Ngodhe), Lake Victoria mainland (e.g., Suba, Kisumu, and Kombewa, KE), East Africa (e.g., Kilifi, KE; Muleba, TZ; Nachingwea, TZ; Apac, UG), Central Africa (e.g., Cameroon), West Africa (e.g., The Gambia and Mauritania)

Supplementary Table S4. Non-synonymous single nucleotide polymorphisms (SNPs) in known drug resistance genes. Known resistance-conferring SNPs highlighted in **bold**.

Gene	Position	Ref	Alt	Mutation	LV islands* MAF (n = 29)	East Africa* MAF (n = 228)	West Africa* MAF (n = 167)	Maximum F_{ST}
Pfcrt	403291	G	T	**D24Y**	**0.071**	**0.078**	**0.000**	**0.099**
	403625	A	C	**K76T**	**0.179**	**0.167**	**0.130**	**0.440**
	404407	G	T	**A220S**	**0.179**	**0.155**	**0.127**	**0.373**
	404836	C	G	**Q271E**	**0.179**	**0.164**	**0.128**	**0.413**
	405362	A	G	**N326S**	**0.000**	**0.0075**	**0.048**	**0.014**
	405596	G	A	A355T	0.000	0.0038	0.000	0.017
	405600	T	C	**I356T**	**0.000**	**0.0075**	**0.180**	**0.340**
	405838	G	T	**R371I**	**0.179**	**0.175**	**0.124**	**0.403**
Pfdhfr	748239	A	T	**N51I**	**1.000**	**0.933**	**0.925**	**0.137**
	748262	T	C	**C59R**	**0.929**	**0.870**	**0.930**	**0.047**
	748410	G	A	**S108N**	**1.000**	**0.995**	**0.930**	**0.129**
	748577	A	T	**I164L**	**0.071**	**0.022**	**0.000**	**0.089**
Pfdhps	549256	A	G	N294S	0.000	0.002	0.000	0.005
	549685	G	C	G437A	0.000	0.120	0.213	0.298
	549993	A	G	**K540E**	**1.000**	**0.858**	**0.300**	**0.944**
	550117	C	G	A581G	0.036	0.015	0.000	0.100
Pfmdr1	957908	G	C	E7Q	0.036	0.005	0.000	0.095
	957990	A	G	K34R	0.000	0.005	0.125	0.006
	958145	A	T	**N86Y**	**0.000**	**0.129**	**0.244**	**0.153**
	958440	A	T	**Y184F**	**0.500**	**0.374**	**0.155**	**0.129**
	958484	A	T	T199S	0.036	0.012	0.000	0.167
	959307	A	G	N473S	0.000	0.003	0.000	0.011
	959399	A	T	N504Y	0.000	0.003	0.000	0.011
	959991	C	A	S701Y	0.000	0.003	0.000	0.011
	960258	C	G	T790S	0.000	0.003	0.000	0.011
	960404	A	G	I839V	0.000	0.002	0.000	0.005
	960702	T	A	**F938Y**	**0.036**	**0.076**	**0.222**	**0.026**
	961481	C	A	Q1198K	0.000	0.002	0.000	0.005
	961625	G	T	**D1246Y**	**0.071**	**0.113**	**0.312**	**0.037**
Pfk13	1725266	C	A	A578S	0.036	0.012	0.000	0.005
	1726234	C	T	R255K	0.071	0.027	0.063	0.051
	1726239	C	A	M253I	0.000	0.003	0.000	0.010
	1726349	T	G	N217H	0.000	0.007	0.000	0.008

	1726431	T	A	K189N	0.000	0.012	0.343	0.011
	1726454	A	T	S182T	0.036	0.017	0.000	0.004
	1726592	G	T	H136N	0.000	0.005	0.031	0.006
	1726652	A	T	L116I	0.000	0.003	0.000	0.010
	1726663	C	T	G112E	0.000	0.003	0.125	0.006
	1726676	T	C	K108E	0.000	0.002	0.000	0.005
	1726711	G	T	P96Q	0.036	0.003	0.000	0.005
	1726933	C	T	R22K	0.000	0.002	0.000	0.005
Pfap2mu	718250	G	C	G99A	0.000	0.003	0.000	-0.010
	718333	G	C	V127L	0.000	0.002	0.111	-0.009
	718391	G	A	R146K	0.000	0.033	0.388	0.025
	718433	G	A, C	**S160N/T**	**0.178, 0.000**	**0.170, 0.004**	**0.380, 0.000**	**0.005, 0.001**
	718550	A	C	K199T	0.000	0.072	0.125	0.018
	718969	C	G	R339G	0.000	0.003	0.000	0.010
	719007	A	T	K351N	0.000	0.002	0.000	0.005
	719265	C	A	F437L	0.000	0.019	0.000	0.051
	719288	A	C	N445T	0.000	0.002	0.000	0.005
	719380	T	G	S476A	0.000	0.008	0.000	0.036

MAF = minor allele frequency; Ref = Reference allele; Alt= Alternative allele; *Lake Victoria (LV) islands (e.g., Mfangano and Ngodhe), East Africa (e.g., Kilifi, KE; Muleba, TZ; Nachingwea, TZ; Apac, UG), West Africa (e.g., The Gambia and Mauritania)

Supplementary Table S5. Genes of interest with SNPs showing selection pressure in the population (iHS value ($-\log_{10}[1 - 2 \mid \Phi_{iHS} - 0.5 \mid]$) > 4).

Population	Gene ID	Gene Function
**Mfangano**	*PF3D7_0809600*	Peptidase family C50; invasion of host cells
	PF3D7_1133400	Apical membrane antigen 1 (PfAMA1); immune evasion against inhibitory antibodies
	PF3D7_1035400	merozoite surface protein 3 (MSP3); generate host antibody response
**Lake Victoria Islands***	*PF3D7_0809600*	Peptidase family C50
	PF3D7_0808200	Plasmepsin X (PMX); parasite egress and invasion
	PF3D7_1035400	MSP3
	PF3D7_1133400	PfAMA1
**Lake Victoria Mainland***	*PF3D7_0808200*	PMX
	PF3D7_1035400	MSP3
	PF3D7_1301700	CX3CL1-binding protein 2 (CBP2)
	PF3D7_1337800	calcium-dependent protein kinase 5 (CDPK5); regulates parasite egress from erythrocytes
**East Africa***	*PF3D7_0104300*	ubiquitin carboxyl-terminal hydrolase 1 (UBP1)
	PF3D7_0103900	parasite-infected erythrocyte surface protein (PIESP15)
	PF3D7_1035400	MSP3
	PF3D7_1301700	CBP2
**Central Africa***	*PF3D7_0104300*	UBP1
	PF3D7_0103900	PIESP15
	PF3D7_1035400	MSP3
**West Africa***	*PF3D7_0709700*	prodrug activation and resistance esterase (PARE); pepstatin resistance
	PF3D7_0709000	chloroquine resistance transporter (CRT)
	PF3D7_1035400	MSP3
	PF3D7_1301700	CBP2

*Lake Victoria islands (e.g., Mfangano and Ngodhe), Lake Victoria mainland (e.g., Suba, Kisumu, and Kombewa, KE), East Africa (e.g., Kilifi, KE; Muleba, TZ; Nachingwea, TZ; Apac, UG), Central Africa (e.g., Cameroon), West Africa (e.g., The Gambia and Mauritania)

Supplementary Table S6. Cross-population analysis of selection pressure on genes of interest within the Lake Victoria (LV) region (XP-EHH (-$\log_{10}$[*p*-value]) > 5).

Population*	Gene ID	Gene Function
**LV islands vs LV mainland**	*PF3D7_0808200*	PMX
	PF3D7_1036300	Duffy binding-like merozoite surface protein 2 (DBLMSP2); A conserved multi-gene family associated with inducing cross-reactive antibodies against *P. falciparum*
**LV islands vs East Africa**	*PF3D7_1036300*	DBLMSP2
	PF3D7_1216600	Cell traversal protein for ookinetes and sporozoites (CelTOS); a conserved antigen with protective potential
	PF3D7_0808200	PMX
**LV islands vs West Africa**	*PF3D7_0808200*	PMX
**LV mainland vs West Africa**	*PF3D7_0709700*	PARE
	PF3D7_0709000	chloroquine resistance transporter (CRT)
	PF3D7_0810800	hydroxymethyldihydropterin pyrophosphokinase-dihydropteroate synthase (DHPS);
	PF3D7_0811300	CCR4-associated factor 1 (CAF1); egress and invasion protein
**East Africa vs LV islands**	*PF3D7_0808200*	PMX
	PF3D7_1035400	MSP3
**East Africa vs LV mainland**	*PF3D7_0810800*	DHPS
	PF3D7_0811300	CCR4-associated factor 1 (CAF1)
	PF3D7_1301700	CBP2
**Central Africa vs LV islands**	*PF3D7_0808200*	PMX
**Central Africa vs LV mainland**	*PF3D7_0810800*	DHPS
	PF3D7_1035400	MSP3
	PF3D7_1224000	GTP cyclohydrolase 1 (GCH1); antifolate susceptibility

*Lake Victoria islands (e.g., Mfangano and Ngodhe), Lake Victoria mainland (e.g., Suba, Kisumu, and Kombewa, KE), East Africa (e.g., Kilifi, KE; Muleba, TZ; Nachingwea, TZ; Apac, UG), Central Africa (e.g., Cameroon), West Africa (e.g., The Gambia and Mauritania)

Supplementary Figure S1. Cumulative genome-wide admixture ancestry proportions for *P. falciparum* populations across the African continent. (**A**) Cumulative percentages of ancestry per region where K is estimated to be 5. (**B**) Cumulative percentages of ancestry per country where K is estimated to be 5.

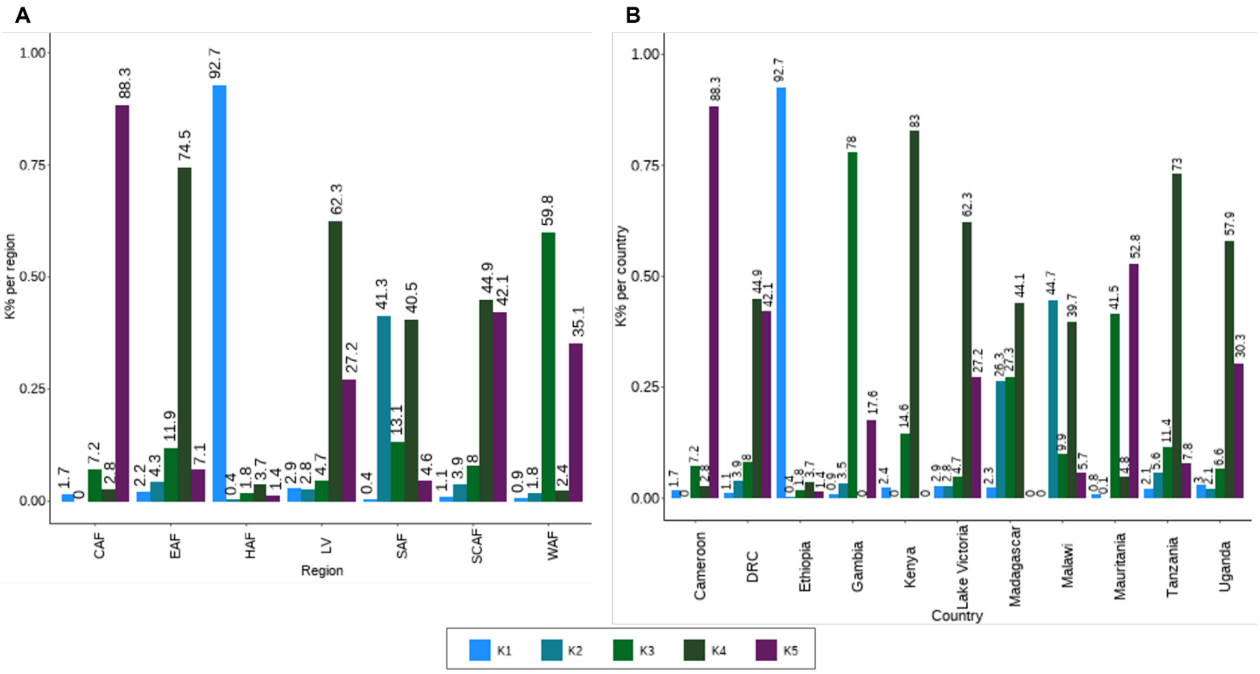

Supplementary Figure S2. Identity by decent (IBD) fractions along each chromosome in *P. falciparum* isolates from (A) Kenya and (B) regional populations across the African continent (e.g. Lake Victoria mainland*, Lake Victoria islands*, Central Africa*, East Africa*, and West Africa*.

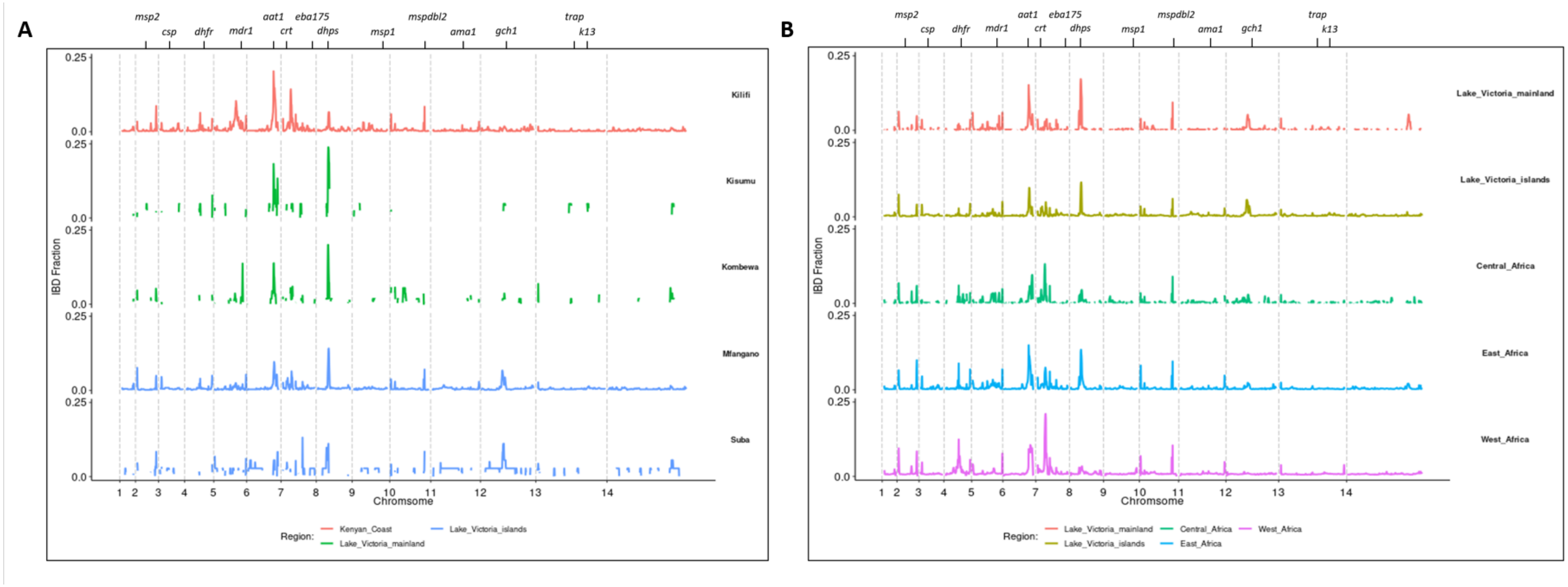

*Lake Victoria islands (e.g., Mfangano and Ngodhe), Lake Victoria mainland (e.g., Suba, Kisumu, and Kombewa, KE), East Africa (e.g., Kilifi, KE; Muleba, TZ; Nachingwea, TZ; Apac, UG), Central Africa (e.g., Cameroon), West Africa (e.g., The Gambia and Mauritania)

Supplementary Figure S3. Signatures of positive selection in Lake Victoria isolates and East African populations. Analysis of haplotype structure to determine genomic regions responding to natural or artificial selection. (**A**) SNPs under selective pressure in Mfangano island isolates with an integrated haplotype score (iHS) >4.0 ($(-\log_{10}[1 - 2 \mid \Phi_{iHS} - 0.5 \mid]) > 4.0$). (**B**) SNPs in Suba District isolates with an iHS value > 4.0. (**C**) SNPs in Lake Victoria isolates with an iHS value > 4.0. (**D**) Cross-population selective pressures identified by comparing SNPs in Mfangano isolates with isolates from Suba District; significant SNPs determined by an *XP-EHH* value > 5.0 ((-log10[p-value]) > 5.0). (**E**) SNPs with an *XP-EHH* value > 5.0 comparing isolates from Lake Victoria with the Lake Victoria mainland. (**F**) SNPs with an *XP-EHH* value > 5.0 comparing Lake Victoria islands with East Africa.

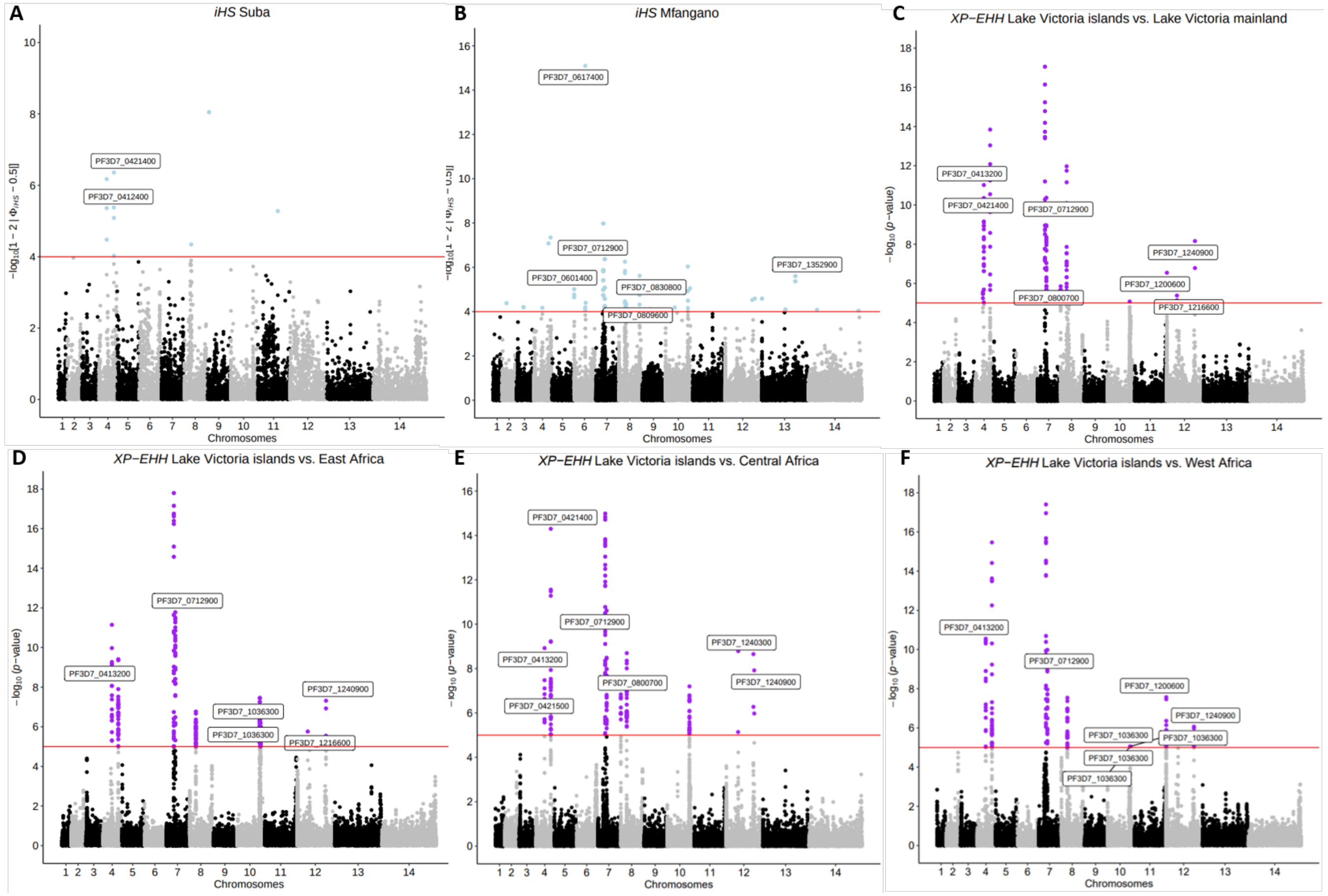

Supplementary Figure S4. Sampling sites and the corresponding number of isolates within the Kenyan region of Lake Victoria. *Total number of Kenyan isolates includes publicly available data from the Pf3K dataset.

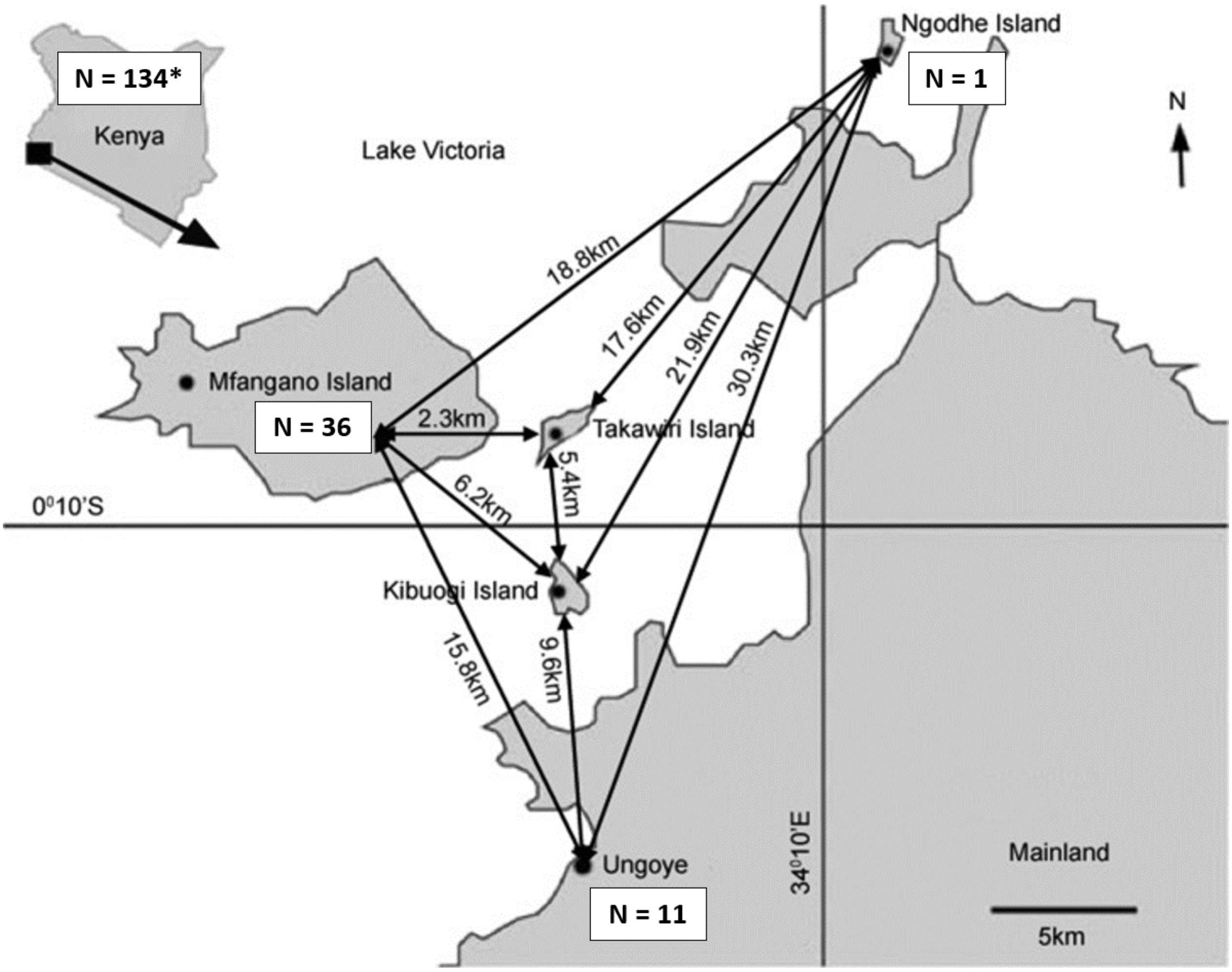

Supplement: Supplementary file 1 — Supplementary Information. [file 41598_2021_99192_MOESM1_ESM.pdf]
